# Differential Network Analyses of Alzheimer's Disease Identify Early Events in Alzheimer's Disease Pathology

**DOI:** 10.1155/2014/721453

**Published:** 2014-07-23

**Authors:** Jing Xia, David M. Rocke, George Perry, Monika Ray

**Affiliations:** ^1^Department of Mathematics, University of California, Davis, CA 95616, USA; ^2^Division of Biostatistics, School of Medicine, University of California, Davis, One Shields Avenue, Davis, CA 95616, USA; ^3^Department of Biology, University of Texas, San Antonio, TX 78249, USA; ^4^Neurosciences Institute, University of Texas, San Antonio, TX 78249, USA

## Abstract

In late-onset Alzheimer's disease (AD), multiple brain regions are not affected simultaneously. Comparing the gene expression of the affected regions to identify the differences in the biological processes perturbed can lead to greater insight into AD pathogenesis and early characteristics. We identified differentially expressed (DE) genes from single cell microarray data of four AD affected brain regions: entorhinal cortex (EC), hippocampus (HIP), posterior cingulate cortex (PCC), and middle temporal gyrus (MTG). We organized the DE genes in the four brain regions into region-specific gene coexpression networks. Differential neighborhood analyses in the coexpression networks were performed to identify genes with low topological overlap (TO) of their direct neighbors. The low TO genes were used to characterize the biological differences between two regions. Our analyses show that increased oxidative stress, along with alterations in lipid metabolism in neurons, may be some of the very early events occurring in AD pathology. Cellular defense mechanisms try to intervene but fail, finally resulting in AD pathology as the disease progresses. Furthermore, disease annotation of the low TO genes in two independent protein interaction networks has resulted in association between cancer, diabetes, renal diseases, and cardiovascular diseases.

## 1. Introduction

Late-onset Alzheimer's disease (LOAD) is a multigene neurodegenerative disorder of the brain and is the commonest form of dementia. Pathologically AD is characterized by the presence of neurofibrillary tangles (NFT) in the neurons and the deposition of amyloid-*β* (A*β*) plaques. Several processes have been associated with AD, such as inflammation, loss of neurons, synaptic pathology, calcium dysregulation, cholesterol synthesis, reentry into the cell cycle, and oxidative stress. Certain brain regions have shown increased susceptibilities to the pathological and metabolic characteristics of AD [1–5]. Analyzing the variations in the transcriptome of the affected brain regions to identify the differences in the biological pathways or processes perturbed in AD can lead to greater insight into its pathogenesis and progression.

**Figure 1:**
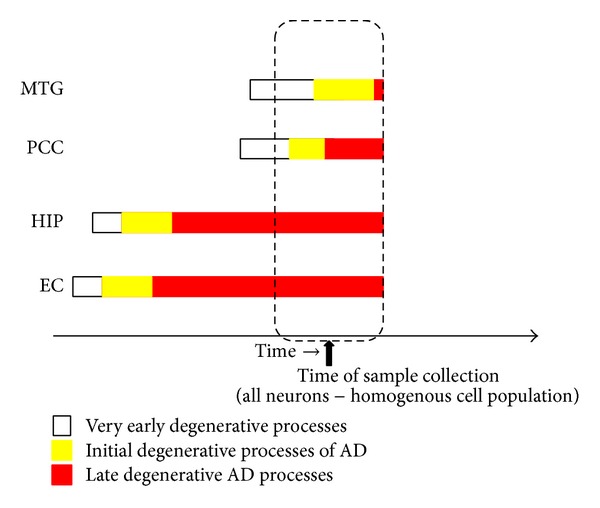
Sample collection and degenerative processes. This figure shows how different brain regions (EC: entorhinal cortex; HIP: hippocampus; PCC: posterior cingulate cortex; MTG: middle temporal gyrus) get affected with degenerative processes over time. The figure is not drawn to scale. Exactly when each set of degenerative processes (as indicated by the white, yellow, and red colors) begins in the different brain regions and how long they last in each region is unknown. This figure shows the logic used in this analysis—that when tissue is collected at the time shown, the PCC and MTG regions which get affected much later in Alzheimer's will have initial or very early degenerative processes as compared to the HIP and EC. The late stage processes will slowly dominate the brain region and, therefore, it will be difficult to identify early AD processes with statistical significance in the EC and HIP.

Alzheimer's disease affects multiple brain regions but begins in the entorhinal cortex (EC) and hippocampus (HIP) [6]. Other brain regions such as the middle temporal gyrus (MTG) and the posterior cingulate cortex (PCC) get affected later during the course of the disease [6, 7]. In the data analyzed in this report, samples (neurons only) that were collected from the PCC and MTG would be from brains that already have AD in the EC and HIP. As shown in Figure 1, the amount of degeneration in the PCC and MTG may not be as severe as in the EC and HIP.

**Figure 2:**
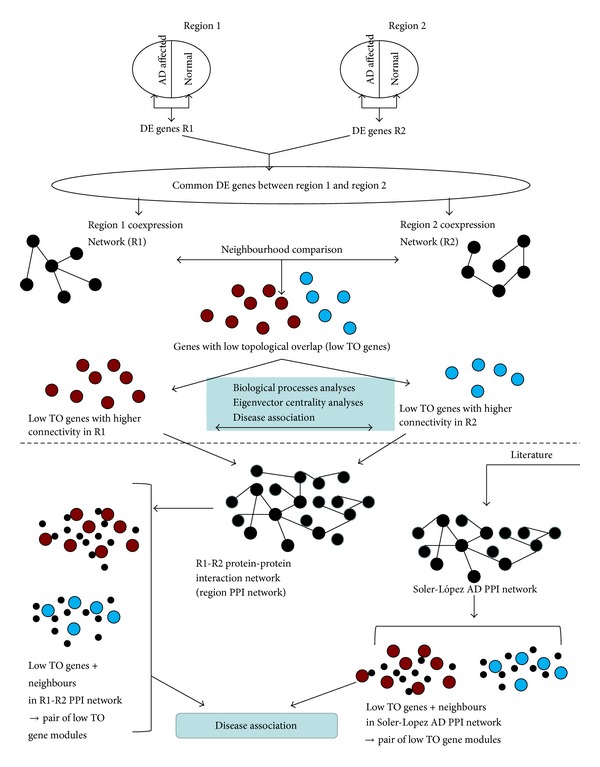
Analyses flowchart. The top half of the figure (above the dashed line) shows how the genes with low topological overlap (low TO genes) are selected and then analyzed for association with biological processes and diseases. The bottom half of the figure shows all the comparisons with the protein-protein interaction (PPI) networks. The region PPI network was created using our set of low TO genes as seed nodes. Since there were six regional comparisons, there resulted six region PPI networks. For each of the 6 regional comparisons, a pair of low TO gene modules was created. One low TO gene module contained the low TO genes that had a higher connectivity in a certain region (say R1) and their direct neighbors in the R1-R2 region PPI network and the other low TO gene module contained the low TO genes that had a higher connectivity in the other region (R2) and their direct neighbors in the R1-R2 region PPI network. The Soler-López AD PPI network was reconstructed from the report by Soler-López et al. The low TO genes from our analyses were mapped onto the Soler-López AD PPI network for the creation of the pair of low TO gene modules. The creation of the pair of low TO gene modules was the same as before except that this time the neighbors were selected from the Soler-López AD PPI network.

In this study, we employ a novel differential network topology method to examine these four AD affected brain regions for differences in biological processes affected in the disease. We use graph theory methods to analyze the low TO nodes (genes) in a gene coexpression network corresponding to different brain regions affected in AD. The coexpression network was built from differentially expressed genes identified from samples consisting of homogeneous cell population of neurons. We extract the low TO genes from gene coexpression networks, which represent different brain regions, to identify the biological processes that may be involved in early AD. We postulate that these selected low TO genes may contribute to the difference in the response to the disease in different brain regions. Furthermore, we identified the diseases associated with low TO genes and neighborhoods in two separate protein-protein interaction (PPI) networks. Finally, we suggest a possible sequence of biological phenomena that may characterize AD progression. Our goal is to leverage insights to better understand processes involved in early Alzheimer's that may be the cause of neurodegeneration. The complete analysis approach is shown in Figure 2.

## 2. Materials and Methods

### 2.1. Expression Data

For our analyses we used microarray (Affymetrix Human Genome U133 Plus 2.0) expression data obtained via laser captured microdissection and data from different brain regions that are either histopathologically or metabolically relevant to Alzheimer's disease (AD) (GEO accession number GSE5281) [1]. Brain samples were from clinically classified late-onset AD patients and normal controls of either sex. The mean age at death was 79.9 ± 6.9 years. Data included expression profiles from the entorhinal cortex (EC; Brodmann area (BA) 28 and 34), hippocampus (HIP; CA1 region), middle temporal gyrus (MTG; BA 21 and 37), and posterior cingulate cortex (PCC; BA 23 and 31). The EC and HIP are affected by intracellular neurofibrillary tangles, and the MTG and PCC are preferentially affected by metabolism and extracellular amyloid-*β* plaques [1]. Alzheimer's affected subjects had a Braak stage ranging from III to VI [6] with a Consortium to Establish a Registry for Alzheimer's Disease (CERAD) neuritic plaque density of moderate or frequent. The expression data was obtained from approximately 500 non-tangle-bearing cortical pyramidal neurons from AD afflicted subjects for direct comparison with non-tangle-bearing neurons from neurologically healthy individuals of the control group. The data consisted of 13 control subjects and 10 AD individuals for EC, 13 control subjects and 10 AD individuals for HIP, 12 control subjects and 16 AD individuals for MTG, and 13 control subjects and 9 AD individuals for PCC. For further information regarding this data, please refer to [1].

Probe sets were normalized using the GC robust multiarray average (GC-RMA) algorithm and processed for differential expression by using the two-class significance analysis of microarrays (SAM) [8]. SAM uses a modified *t*-statistics method to identify DE genes. Within each brain region, AD affected subjects and normal controls were compared to identify differentially expressed (DE) genes. Differentially expressed (DE) genes refer to those genes that have a difference in their mRNA expression level between the affected and control groups. We used DAVID to perform functional annotation clustering (similarity threshold = 0.80) based on diseases associated, if any, with the genes [9].

### 2.2. Coexpression Networks Creation

Gene coexpression networks were built by connecting genes whose pairwise expression similarity using the Pearson correlation coefficient (PCC) was at or above a threshold *t*. For two genes to be considered as coexpressed, their expression profiles need to satisfy at least one of the following conditions: (1) their correlation coefficient is higher than 0.3, and one gene is ranked as the top 3 most correlated genes of the other; (2) the correlation coefficient between them is higher than a user defined Pearson correlation coefficient threshold *t* (*t* = 0.7 in all the networks constructed here) and one gene is within the top 50 most correlated genes of the other [10]. The coexpression network construction is described in detail in [10]. The main reason for choosing a small PCC cut-off of 0.3 is that as the PCC increases the number of nodes with no links also increases (see Figure  1 in [10]). We constructed a coexpression network with no isolated nodes. Also, we chose to connect the nodes with PCC of 0.3 only to 3 nodes since the median number of links is very high for nodes with a low PCC and that would result in too many unnecessary links with low PCC (see Figure  1 in [10]). This approach resulted in a sparse, fully connected, unweighted, and undirected coexpression network. The coexpression network is represented as a binary adjacency matrix with 0 referring to no link between two genes and 1 corresponding to a link between the genes. This network method has had other successful applications [11–14].

A coexpression network was constructed for a brain region under investigation using the differentially expressed (DE) genes that were common to two brain regions and using the samples (control and affected) of that specific region (see Figure 1 (top half) and Figure 2). This approach resulted in six comparisons of brain regions: EC with HIP (EC-HIP), EC with MTG (EC-MTG), EC with PCC (EC-PCC), HIP with MTG (HIP-MTG), HIP with PCC (HIP-PCC), and PCC with MTG (PCC-MTG), as shown in Figure 2.

### 2.3. Topological Overlap between Coexpression Networks

Let coexpression networks* network 1* and* network 2* correspond to brain regions 1 and 2, respectively. Since* network 1* and* network 2* were built using the common DE genes between regions 1 and 2, the networks have the same set of nodes/genes but different connections. A node/gene in the network is denoted by *gene*_*i*_ where *i* = 1,2,…, *m* and *m* is the total number of nodes in the network.

The sets of genes connected to *gene*_*i*_ in* network 1* and* network 2* are *X*_*i*1_ and *X*_*i*2_, respectively. The topological overlap (TO) for *gene*_*i*_ between* network 1* and* network 2* is

(1) $$\begin{array}{c} \text{T}{\text{O}}_{i}=\frac{\left|X_{i1}⋂X_{i2}\right|+1}{\max\left(\left|X_{i1}\right|,\left|X_{i2}\right|\right)}\cdot \frac{1}{\sqrt{\left|X_{i1}\right|\cdot \left|X_{i2}\right|}},\\ \frac{1}{d_{i}^{2}}\leq \text{T}{\text{O}}_{i}\leq \frac{d_{i}+1}{d_{i}^{2}},\;\text{where}\;\;d_{i}=\max\left(\left|X_{i1}\right|,\left|X_{i2}\right|\right). \end{array}$$

The above formula is a modification of our previous topological overlap formula described in [11, 12]. The formula ranks genes based on its (i) neighborhood size and (ii) difference between neighborhoods in the two regional coexpression networks. We wanted to identify genes that have not only low neighborhood overlap but also large neighborhood size.

There are nine possible scenarios of neighborhood overlap as shown in Figure 4. Scenarios 1, 2, and 6 occur when the neighborhoods of a gene in both regional networks are large and they have either zero (scenario 1), small (scenario 2), or large/complete (scenario 6) overlap of their neighbors. When the gene has a large neighborhood in one network and a small one in the other, the possible kinds of overlap are zero (scenario 3), small (scenario 4), and large/complete overlap (scenario 5). In scenarios 7, 8, and 9, when both neighborhoods of the gene are small, they could have either zero (scenario 7), small (scenario 8), or large/complete overlap (scenario 9). A gene with a large difference in its neighborhood size and low overlap (scenario 3 and scenario 4) or one with large neighborhoods and low overlap (scenarios 1 and 2) is very interesting, because it implies that this gene may be active and may play a very different role in the two regions. A gene is ranked the highest if it has a large neighborhood but no overlap of neighborhoods. The new low TO formula resulted in better identification of relevant biological processes in the different regions.

Genes were ranked according to their topological overlap in ascending order. Brain region comparison was done using top 10% of ranked genes (see Section 3). Since the conditions are similar (both are AD affected, with high overlap of the common DE genes), there is bound to be more similarities, than differences, between the different networks. We chose 10% as there would be a small number of genes contributing to the difference between the regions since the condition, although similar, is still not identical. Genes with other values of topological overlap, if properly justified, can also be considered [11]. Comparisons against 100 random networks (random additions or deletions of edges in the network while keeping the total number of edges in the entire network equal to the original network) using *t*-statistics were made to assess the significance of low TO genes. All sets of low TO genes had significance *P* < 0.05 compared to random networks.

Biological analysis and interpretation of genes of interest was performed by identifying significant biological processes or pathways. Statistically significant biological pathways were identified using the well annotated GeneGo MetaCore database [15], which is a commercial tool. MetaCore is based on a proprietary manually curated database of human protein-protein, protein-DNA, and protein compound interactions, metabolic and signaling pathways, and the effects of bioactive molecules in gene expression [15]. We upload a set of genes into GeneGo MetaCore module and GeneGo checks to find which pathways are significant with a FDR = 0.05. It compares the user's uploaded set of genes with the set of genes/proteins stored in their pathways database. Significance (*P* values) in GeneGo of a biological pathway is evaluated based on the size of the intersection between user's dataset and set of genes/proteins corresponding to a network module/pathway in question. Thorough details of the significance calculation are provided in [16, 17].

### 2.4. Eigenvector Centrality Calculation

Centrality is a function *C* which assigns a numeric value to every vertex *v* in a graph *G*, say *C*(*v*). In graph *G*, a vertex *u* is more important than a vertex *v* if and only if *C*(*u*) > *C*(*v*). In social networks, centrality is used to find the most “influential” or “central” nodes. Among different kinds of centrality, eigenvector centrality has been used to study epidemics as it is the best measure of spreading power in a network [18]. An eigenvector *ϕ* and associated eigenvalue *λ* are defined by

(2) $$\begin{array}{c} \lambda \phi =A\phi , \end{array}$$

where *A* is the adjacency matrix of the graph. If *ϕ* is an eigenvector then so is *κϕ* for any *κ* ≠ 0. Often *ϕ* is defined to have unit length and is then, in general, unique up to sign.

In an undirected connected graph *G* = (*V*, *E*), *V* is the set of vertexes/nodes with |*V*| = *n* and *E* is the set of edges. Let *A* be its adjacency matrix, where *a*_*ij*_ = 1, if (*v*_*i*_, *v*_*j*_) ∈ *E*; *a*_*ij*_ = 0, otherwise. Bonacich [19] defines eigenvector centrality of a vertex *v*_*i*_ as

(3) $$\begin{array}{c} EgC\left(v_{i}\right)=\frac{1}{\lambda }\underset{v_{j}\in V}{\sum }a_{ij}EgC\left(v_{j}\right); \end{array}$$

equivalently, let *c* = (*EgC*(*v*_1_),…, *EgC*(*v*_*n*_)),

(4) $$\begin{array}{c} Ac=\lambda c. \end{array}$$

It has been shown that, for an adjacency matrix of an undirected graph, there exists a nonnegative eigenvector (all entries are nonnegative). The entities of this nonnegative eigenvector are centrality scores of the vertexes/nodes in *G*. If we normalize *c* = (*EgC*(*v*_1_),…, *EgC*(*v*_*n*_)) under Euclidean norm such that its Euclidean norm is $||c||=\sqrt{2}$, *EgC*(*v*_*i*_) can be used for comparison among networks of different sizes [20]. In this paper, Euclidean normalized eigenvector centrality was calculated using the the function* evcent()* in the R package* igraph*.

We computed the eigenvector centrality scores for (i) low TO genes between brain regional networks; (ii) low TO genes between affected and control networks within each brain region (coexpression networks are built on DE genes between the affected and control); and (iii) DE genes between affected and control within one brain region. Construction of the coexpression networks in (i) is explained in “Coexpression Networks Creation.” The coexpression networks in (ii) and (iii) are briefly explained below.

The differentially expressed genes between affected and control samples within each region were used to build an “affected” and a “control” coexpression network for that brain region (a total of 8 coexpression networks). Our topological similarity measure was applied to select genes with low topological overlap (TO) between affected and control coexpression networks within a brain region. Four sets of low TO genes were obtained for four brain regions. Then the eigenvector centrality values of these low TO genes in the affected and control networks were computed. These results would aid us in determining if the average eigenvector centrality scores of the low TO genes were higher or lower in the affected network compared to the control. Then the eigenvector centrality scores were computed for the low TO genes obtained by comparing brain regions in order to determine if one region had lower/higher average eigenvector centralities compared to the other. Welch's *t*-test was used to check whether the mean eigenvector centrality score in one group was significantly different from the other and two-tailed *P*-values were noted for all comparisons.

### 2.5. Protein-Protein Interaction Networks Construction

Protein-protein interactions were obtained from the Human Protein Reference Database (HPRD database) (March, 2012) to construct the regional protein-protein interaction network (region PPI network). All genes were converted to their corresponding protein products. Six protein sets from six pairwise regional comparisons were used as seed sets. Full PPI networks were constructed by firstly including direct neighbors of seed proteins and then neighbors of those neighbors until no more extra proteins could be included. Full PPI networks were pruned such that the fewest possible nonseed proteins were included to keep connected seeds in the full PPI networks still connected. The pruning procedure is briefly explained below.

**Phase 1 .**

For all connected seeds, include all extra nodes on the shortest paths connecting those seeds.

**Phase 2 .**

For each pair of connected seeds and each shortest path connecting them, count the number of seeds on this shortest path. Find the maximum for one pair of connected seeds, denoted by *M*. Exclude nonseed nodes from the shortest paths that have less than *M* seeds.

**Phase 3 .**

For each shortest path *p* passing through the first two pruning phases, count the number of times its nonseed node *V*_*i*_ appears on a shortest path connecting two seeds, denoted by *n*_*i*_. The frequency of *p* is defined as *N*_*p*_ = sum_*V*_*i*_ *on*⁡  *p*_ *n*_*i*_. For each pair of seeds, prune nonseed nodes on the shortest paths with *N*_*p*_ < max_*p*  connecting  seeds_(*N*_*p*_).

Since all protein-protein interactions in HPRD are undirected relations, the regional PPI networks in this study are undirected networks. Supplemental information about the detailed pruning procedure and sizes of PPI networks before and after pruning is available upon request.

### 2.6. Alzheimer's Disease Protein Network Construction

The largest network of protein interactions related to Alzheimer's was reported by Soler-López et al. [21] and referred to as “AD PPI network.” In Soler-López et al.'s study [21], 12 known AD associated genes (seeds) were selected. Through yeast two-hybrid (Y2H) matrix screen and yeast two-hybrid (Y2H) library screen, 200 interactions with high confidence level between 74 nodes were detected, including seed-seed, seed-candidate, and candidate-candidate interactions. A high confidence network (HC network) regarding AD was constructed based on the 200 interactions. This HC network was merged with direct interactors of 12 AD seeds from IntAct, DIP, MINT, and HPRD. The AD network contains 1704 nodes and 5881 interactions and was stored as a binary adjacency matrix. In our analyses, the official gene symbols were converted to UniprotKB accession numbers and then to Entrez gene IDs for functional annotation analysis in DAVID.

## 3. Results

### 3.1. Significant Biological Processes of Genes with Low Topological Overlap

Figure 1 is critical to our analyses and interpretation of the results. Through the figure we attempt to show that, in an AD affected individual's brain, the entorhinal cortex (EC) and the hippocampus (HIP) get affected much before the posterior cingulate cortex (PCC) and the middle temporal gyrus (MTG). Furthermore, when a region gets affected with the disease, the initial biological processes are different from the final biological processes that lead to neurofibrillary tangles and extracellular neuritic plaques associated with AD. The knowledge of the early events in AD can help in early AD detection, therapeutic intervention, and drug development. We show in Figure 1 that when samples are collected from affected PCC and MTG regions in AD affected brains, the amount of degeneration or the kind of biological processes may be related to early AD as opposed to late AD.

**Figure 3:**
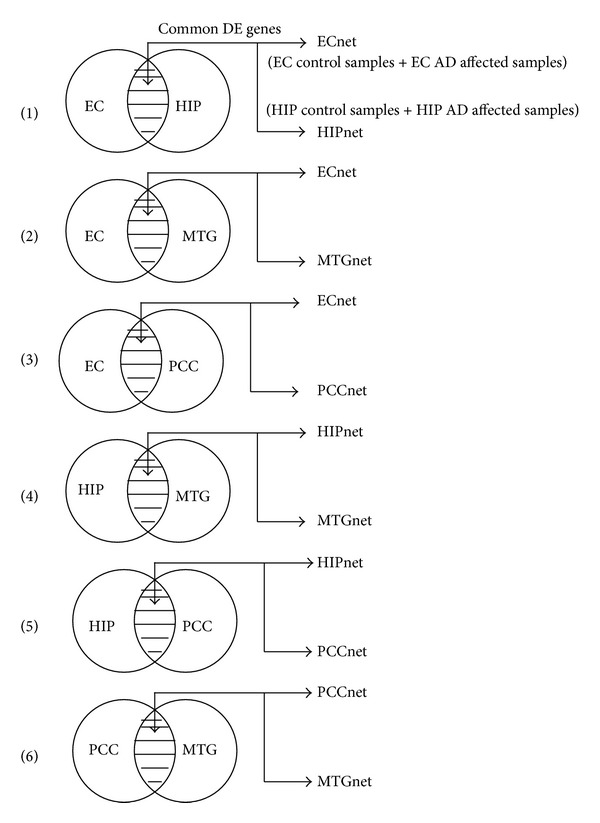
Regional coexpression networks construction diagram. Gene coexpression networks built from sets of differentially expressed genes. EC refers to the entorhinal cortex, HIP is hippocampus, MTG is the middle temporal gyrus, and PCC is posterior cingulate cortex. For each of the 6 comparisons, the coexpression network was built using the common differentially expressed genes between the two regions and the samples from that specific region. For instance, when the EC and HIP were being compared, the 2041 common DE genes between EC and HIP were used to construct the ECnet using the control and the AD affected samples from the EC, while the HIPnet was constructed on the same set of 2041 DE genes but the control and AD affected samples were from the HIP. This kind of analyses results in 12 coexpression networks—2 per regional analysis.

**Figure 4:**
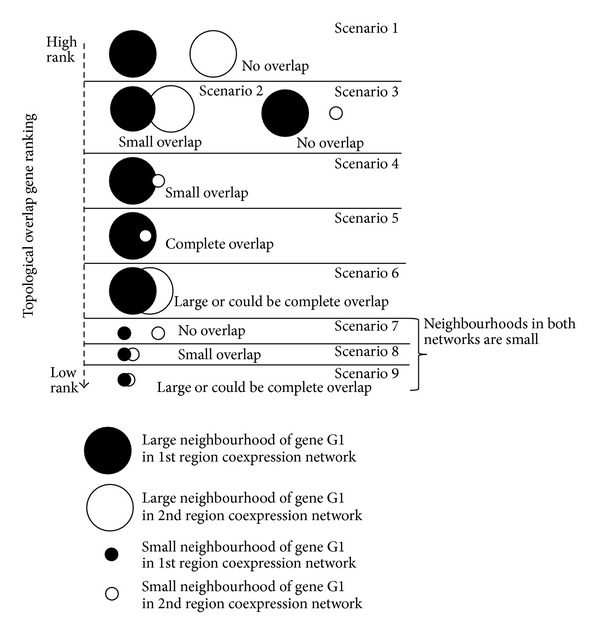
Ranking of low TO genes. Nine possible scenarios of how neighborhoods overlap when considering neighborhood size and neighborhood overlap size of a gene, simultaneously, in two regional coexpression networks. Scenarios 1, 2, and 6 occur if the neighborhood of a gene in both regional networks is large and either they have zero (scenario 1), small (scenario 2), or large/complete (scenario 6) overlap of their direct neighbors. In scenarios 3, 4, and 5, the gene has a large neighborhood in one network and a small neighborhood in the other coexpression network. This also could lead to three possible kinds of overlap: zero (scenario 3), small (scenario 4), or large/complete overlap (scenario 5). Finally, in scenarios 7, 8, and 9, both neighborhoods of the gene are small and could have either zero (scenario 7), small (scenario 8), or large/complete overlap (scenario 9). The scenarios at the top of the figure have a higher rank than the ones at the bottom. Scenarios 1, 2, and 3 have a higher rank because the gene has either (1) a large neighborhood in both regional networks but small/no overlap of its neighbors or (2) large difference of its neighborhood in the two regional coexpression networks and no overlap of its neighbors whatsoever. Scenarios 4 and 5 have a large difference in neighborhoods but small or complete overlap of their neighbors. A gene is ranked the highest if it has a large difference in its neighborhood size and no overlap of its neighbors. This gene is assumed to have high activity (as indicated by the large neighborhood size) yet different biological roles (as indicated by the zero overlap of its neighborhoods) in the two different regions.

The number of differentially expressed (DE) probes per region is shown in Table 1. Six sets of common DE genes (see Section 2) were obtained from six comparisons: (1) entorhinal cortex (EC) and hippocampus (HIP); (2) entorhinal cortex (EC) and posterior cingulate cortex (PCC); (3) entorhinal cortex (EC) and middle temporal gyrus (MTG); (4) hippocampus (HIP) and posterior cingulate cortex (PCC); (5) hippocampus (HIP) and middle temporal gyrus (MTG); (6) posterior cingulate cortex (PCC) and middle temporal gyrus (MTG) (see Figure 3).

Twelve coexpression networks were built as shown in Figure 3. Genes with low topological overlap were identified in each regional comparison. The number of genes with low topological overlap (TO) between different brain regions is shown in Table 2. We derive our conclusions from 2 ideas: (1) that the PCC and MTG get affected later in AD progression after the EC and HIP [7, 22] (hence, studying the PCC and MTG in brains that already have AD affected EC and HIP will result in identifying the biological processes that occur early in AD pathogenesis) and (2) that genes with low overlap of their neighborhoods in the two regional networks are the genes that may contribute to the biological difference between regions in their response to AD [11, 12]. They may invoke either different biological functions (as they are connected to different sets of neighbors) or the same biological pathways to varying degrees [23–26].

We show the significant biological processes for the low TO genes with a higher connectivity (greater number of edges in the coexpression network) in a certain region in Table 3. The significant biological processes of the six sets of low TO genes (without dividing into groups of higher connectivity) are provided as supplemental information that is available upon request. Since the PCC and MTG get affected later in AD progression after the EC and HIP [7, 22], it is probable that the processes present in the PCC and MTG occur early in AD pathogenesis. The EC and HIP have a wide range of processes, such as those involved in response to stress, apoptosis, cell cycle, immune response, postsynaptic membrane assembly, memory and learning, and calcium ion transport. The PCC and MTG seem to be mainly involved in signaling and antiapoptotic pathways. All these processes have been previously associated with AD [27–33]. It is possible that, mainly, defense mechanisms are occurring in the PCC and MTG, although to varying degrees in the two regions. Our results imply that cell cycle and certain signaling pathways, along with immune responses, occur in early AD. These processes include PEDF signaling, delta- and kappa-type opioid receptors signaling via beta-arrestin, glucocorticoid receptor signaling, and WNT signaling pathway.

Pigment epithelium-derived factor (PEDF), also known as serpin F1 (SERPINF1), is a multifunctional secreted protein that has antiangiogenic, antitumorigenic, and neurotrophic functions. PEDF induces PPAR-gamma expression which in turn induces p53, a tumor suppressor gene involved in cell cycle regulation and apoptosis [34, 35]. PEDF is downregulated in hypoxic conditions and by *β*-amyloid when investigated in the retinal cells. It has also been reported to be affected in AD patients [36]. Maarouf and colleagues [36] have reported that PEDF's upregulation in AD brains could be a defense response since PEDF has anti-inflammatory, antioxidant, antithrombotic, and neuroprotective properties, but it could also have a negative function by preventing blood vessel formation leading to neurodegeneration. Downregulated PEDF has been detected in the plasma of AD patients and was identified as a potential biomarker for AD [37]. It was found to be upregulated in the cerebrospinal fluid of AD patients [36]. The glucocorticoid receptor (GR or GCR) is also known as NR3C1. The GR regulates genes controlling the development, metabolism, and immune response. The activated GR complex upregulates the expression of anti-inflammatory proteins in the nucleus or represses the expression of proinflammatory proteins in the cytosol. The glucocorticoid receptor is gaining interest since it has a role in the stress response in the brain. It is implicated in both short- and long-term adaptations seen in response to stressors and may be a key factor to the understanding of psychological disorders [38]. Glucocorticoids have been implicated in aging, AD, and impaired learning when studied in mice and rat brains [39, 40]. Many reports have shown the association of WNT signaling pathway and Alzheimer's disease [41–43]. It has been implicated in the cause of neurodegeneration in AD brains.

Cell cycle reentry by neurons has been reported to be the cause of neuronal death in Alzheimer's [32, 44–46]. It has also been shown that neurons definitely die if they begin cell cycle processes and the cell cycle progression blockers fail to stop it [44]. However, from the results in Table 3, we find that cell cycle processes are not very active in the MTG and PCC regions. This may imply that cell cycle reentry processes are not early events in AD. We do notice some processes in the MTG and PCC that may have a role in learning and memory impairment by disrupting neural plasticity, such as ephrin signaling and glucocorticoid receptor signaling [39, 40, 47]. Specifically, glucocorticoids have been associated with increasing neurotoxicity of reactive oxygen species and oxidative stress in cells [48, 49]. Recently, BRCA1 has been reported to be the cause of reentry into the cell cycle [50]. A very interesting secondary observation was that we did not find BRCA1 to be differentially expressed in the PCC and MTG; however, it was differentially expressed in the HIP and EC regions. It was not selected as a low TO gene, probably because it did not change its behavior across regions. This could mean that BRCA1 does not get expressed until much later in AD progression or probably it only gets expressed in the most vulnerable regions such as the EC and the HIP. This observation warrants further investigation.

Another biological process that seemed significant was oxidative stress. Processes in the PCC and MTG that are indicative of oxidative stress are stress activated MAPK cascade, glucocorticoid receptor signaling, nuclear factor-*κ*B pathway, and regulation of protein tyrosine kinase activity. The fact that oxidative stress may be an early event in AD has been reported in many studies [51–53]. Since we found oxidative stress (OS) as a highlighted process in early AD, we wanted to identify what neurodegenerative biomarkers may have been identified so far for OS. For that purpose, we compared 159 neurodegenerative disease related genes obtained from the KEGG disease database (using keywords neurodegenerative, dementia, aging, and cognitive impairment) with our set of common DE genes between regions (this set of genes was used to build coexpression networks). Four genes—*AARS* (alanyl-tRNA synthetase),* PINK1* (PTEN-inducible kinase 1),* SNCB* (*β*-synuclein) and* UBE1* (ubiquitin-activating enzyme E1)—were present in all six sets of common DE genes. This indicates that all the four regions had these four genes differentially expressed between normal controls and AD affected subjects. Of the four genes, reports have linked PINK1 with OS. The PINK1 gene has been heavily studied in the contest of Parkinson's disease [54–56]. It protects against mitochondrial dysfunction during cellular stress [56]. PINK1 was always downregulated in the MTG compared to the EC, HIP, and PCC, when affected samples from each region were compared against each other. PINK1 deficiency has been associated with impaired respiration and increased production of reactive oxygen species in the mitochondria [53]. This result further confirmed our conclusion from our method that OS was truly an early event in AD.

Based on the results reported here and other researches, we postulate the following sequence of processes in AD:* oxidative stress *→* signaling processes and antiapoptotic processes involved in cell defense *→* reentry into cell cycle and failure of neural plasticity *→* disruptions in postsynaptic activities *→* immune response, apoptotic processes, and multiple other processes activated *→* cell death *→* disruptions in memory and general brain decline*. We think that, initially, the cellular mechanisms are able to balance the stress activated proapoptotic and survival pathways; however, as more cellular machinery disruptions occur, this balance is lost resulting in eventual cell death.

### 3.2. Eigenvector Centrality Scores of Low TO Genes

A study by Goh et al. shows that the vast majority (approximately 78%) of disease genes are nonessential genes, which neither are hub genes themselves nor do they encode hub proteins [57]. Therefore, we did not identify hub genes (nodes with high degree centrality) in our analysis. However, we decided to measure the eigenvalue centrality of the low TO genes (see Section 2.4). A hub gene can have a high eigenvector centrality but not all genes that have high eigenvector centrality will be a hub gene. Differences in centrality across two biological groups have been reported in many scientific papers [58–62]. Specifically, it has been shown that there is a loss of network connectivity in the brains of AD affected individuals [60–62].

Genes with low topological overlap between affected and control gene coexpression networks were identified for each brain region. Then the eigenvector centrality of low TO genes was computed for the affected and control networks. If network A has a larger mean eigenvector centrality than network B, it means that network A has higher connectivity than network B.

From the results in Table 7, we conclude that the low TO genes in the AD affected individuals have a lower average eigenvector centrality score compared to those in normal individuals (control networks). Although in MTG a higher average eigenvector centrality score was obtained for the affected group, this result was determined to not be significant (*P* > 0.05). The eigenvector centrality scores were calculated for all the DE genes in affected and control networks within each region (results in Table 8). However, they were not as statistically significant as the low TO genes. If we take these results to be indicative of the behavior (in terms of eigenvector centrality values) of the low TO genes in affected samples, then we can perform the same analysis across brain regions and determine which region is more affected than the other. Hence, we analyzed the mean eigenvector centrality scores of the low TO genes across brain regions. From Table 9 we can see that the low TO genes in the MTG had higher average eigenvector centrality score compared to those in the other brain regions. This implies that the MTG is the least affected compared to the other three regions. Since we have hypothesized that because the MTG is affected later in AD compared to the EC and HIP the biological processes that are predominant in the MTG are probably the ones that occur in early AD, the results of eigenvector centrality scores give us further confidence that our kind of analysis on low TO genes is fruitful.

### 3.3. Disease Associations of Genes with Low Topological Overlap

So far we have shown that low TO genes are useful in determining the relative disease severity and identifying some interesting biological processes that may occur in early AD. Since we found the PINK1 gene, which is associated with Parkinson's disease, present in our analyses, we wanted to identify the diseases, if any, that were associated with the low TO genes. Using DAVID, we discovered that diseases such as cardiovascular and renal diseases, bone marrow transplantation, stroke, and cancer were highly associated with some sets of low TO genes (Table 4, column 3). Goh et al. have reported that genes that contribute to a common disorder have a greater tendency to interact at the protein level [57]. In order to gain more insights we decided to look into the protein level of the low TO genes. For further clarity, we also separated the low TO gene modules into which coexpression network they had a higher connectivity in and identified the associated diseases with these low TO gene modules (Table 5).

We performed two sets of analyses: (i) analyses of the protein-protein interaction network built using the set of common genes between regions and (ii) analyses of the protein-protein interaction network built by Soler-López et al. [21] (see Figure 1).

### 3.4. Comparison of Regional mRNA Coexpression Networks with Protein-Protein Interaction Networks

Six protein-protein interaction (PPI) networks (EC-HIP, EC-MTG, EC-PCC, HIP-MTG, HIP-PCC, and PCC-MTG) were constructed as described in Materials and Methods. We refer to this PPI network as “region PPI network.” We wanted to identify the diseases associated with the low TO genes and their direct neighbors in the PPI networks (see Figure 1 (below dashed line)).

The diseases associated with the low TO genes only (primary set) are shown in column 3 of Table 4 and the diseases associated with these low TO genes and their direct neighbors (extended set) in the region PPI network are shown in column 2 of Table 4.

For further clarity, the low TO genes identified from the gene coexpression networks were divided into two groups based on in which regional gene coexpression network they had a higher connectivity (see Table 5). Thus, in each regional comparison, a pair of low TO gene “modules” in the PPI network was obtained, which included low TO genes and their corresponding direct and unique neighbors in the region PPI network. For instance, in the EC-HIP PPI network, one low TO gene module consisted of low TO genes (including their direct neighbors in the EC-HIP PPI network) whose connectivity is higher in the EC gene coexpression network and another low TO gene module consisted of low TO genes (and their direct neighbors in the EC-HIP PPI network) whose connectivity is higher in the HIP gene coexpression network. Functional annotation clustering for diseases in DAVID was implemented for the unique proteins in the two modules (common proteins between a pair of low TO modules were removed) in all 6 comparisons. Genetic association disease database and OMIM disease database were used with a similarity threshold set to 0.80.

We refer to the largest protein interaction network related to Alzheimer's from Soler-López et al. [21] as “AD PPI network.” We analyzed this AD PPI network for disease associations of the low TO genes. In each of the 6 regional comparisons, we first identified the set of overlapping low TO genes between the AD PPI network and our set of low TO genes. These sets are shown in column 1 of Table 6.

Then as discussed before, pairs of low TO gene modules were obtained for all the networks. For instance, in the EC-HIP comparison, the two low TO gene modules consisted of low TO genes whose connectivity is higher in the EC or HIP gene coexpression network and their direct neighbors in the AD PPI network. As before, functional annotation clustering for diseases in DAVID was implemented for each pair of low TO gene modules in all 6 comparisons. Both genetic association disease database and OMIM disease database were used. Similarity threshold was set to 0.80. Top four diseases with the lowest *P* values in the clusters with enrichment score >0.80 are reported in Table 6.

As can be noted from Table 4, the association with cardiovascular diseases, diabetes, and renal and cancer related conditions was strong (enrichment score of 0.8 or higher). Among these diseases, AD and vascular dementia were also present. This implies that there may be some disease biomarkers in the set of low TO genes. By comparing the results obtained from the region PPI networks and the AD PPI network, we see that there is a consistency in the results from independent studies. Therefore, analyses by us and others have shown associations between AD and cardiovascular diseases and diabetes [14, 63–68]. Interestingly, the PINK1 gene also has been recently associated with heart failure [69]. From the analyses reported here, it is possible that individuals that already have cardiovascular diseases or diabetes may be predisposed to Alzheimer's.

## 4. Discussion

Relating AD to the fundamental choices of cells as they age is a critical area of research. The history of AD research has been hindered by biased hypothesis driven approaches which have reached their culmination in the recent failure of numerous clinical trials. In our paper, we present a novel data driven approach to dissect AD and provide insights to uncover the initial departure from normal physiology. We developed a novel method for the comparative analysis of gene coexpression networks representing different biological regions. This method is best suited for the analyses of progressive conditions since the changes across time may be subtle in most biological conditions. The low TO formula developed here helps in ranking the genes based on 3 criteria: how much their neighborhoods overlap, the size of the neighborhood, and the difference in neighborhood size.

Systems biology methods like this are in high demand as the differences between many conditions, be they neurodegenerative diseases, brain diseases, different kinds of cancers, different degrees of disease severity, and so forth, are very subtle and cannot be easily highlighted using the usual off-the-shelf clustering or biological pathways identification algorithms. Many studies investigate only the genes that are unique to a condition, in order to analyze how different the conditions are. However, we hypothesize that even the genes that are common between conditions (conditions can be physiological, treatment, or time) can contribute to the differences between conditions either by invoking different biological pathways or by invoking the same biological pathways to varying degrees. Our differential network analysis method is applicable to other studies where a sequence of activities or processes is being determined. For instance, in our time-dependent analysis of low dose ionizing radiation study we were able to show the active biological processes at 3, 8, and 24 hours [12]. Our approach can aid in identifying the few genes that may be the key players in the specific condition and, therefore, potential biomarkers or therapeutic targets for that condition.

Due to the oxidative stress hypothesis in AD, therapies involving antioxidants are obvious. However, clinical trials with antioxidants—vitamin E, vitamin C, and coenzyme Q10 (CoQ10)—have failed [53]. This is probably due to late therapeutic intervention. One idea would be to study the effects of antioxidants on different AD affected brain regions in animal models. The comparison of regions can be done using our method of analyses.

Although ours is not the first study to conclude that oxidative stress may be an early event in AD, it is the first that has arrived at this conclusion using an unbiased means of analyses of high throughput genomic data. Our suggestion of the possible sequence of phenomenon in AD is based on the results obtained in our analyses. Along with increased oxidative stress, we think abnormal lipid metabolism may also be an early event in AD pathology. Research investigating whether lipid metabolism is an early event in AD pathogenesis is necessary. The system level observations obtained in this study can have multiple alternative explanations as the processes present in early AD and neuronal degeneration are not fully understood. However, the results allow us to posit testable hypotheses for further investigation. Our results show that since BRCA1 gets significantly expressed in the EC and HIP, but not in the MTG and PCC, and it has been reported to make neurons begin cell cycle, cell cycle reentry is not an early event. Therefore, our results contradict the reports that cell cycle is an early event. It precedes amyloid-*β* plaques and neurofibrillary tangles; however, it is not what triggers the cascade of neurodegeneration in AD.

We chose only to use the common DE genes in the construction of the PPI network as we wanted to be able to compare the results from our coexpression network analyses. We had indications from gene analysis (DE genes or the common DE genes between regions) that certain diseases were overrepresented, such as cardiovascular diseases, diabetes, and cancer. Moreover, it is believed that posttranscriptional modifications and protein interactions can provide a better understanding of a condition as compared to only mRNA expression profiles. This is why we wanted to include PPI networks but restrict them to include only our set of seed genes as much as possible, since our set of seed genes were selected based on AD samples and were, therefore, AD relevant (kind of create an AD PPI network based on our set of DE genes). Else, PPI networks can have 10,000 or more nodes/proteins and would no longer be restricted to AD analyses. In our report we also showed links to other diseases by analyzing the neighborhood of the low TO genes in two independent protein interaction networks. This could imply that certain diseases may predispose an individual to Alzheimer's disease. We have shown links to cardiovascular diseases in our previous report involving gene module detection and transcription factor identification in AD microarray data [14]. Based on such evidence, we believe large randomized trials should be conducted on investigating whether diseases such as diabetes and cardiovascular diseases predispose an individual to AD.

## Figures and Tables

**Table 1:** Number of differentially expressed (DE) genes and associated false discovery rate (FDR) in the four brain regions.

Region	Number of DE genes (FDR)
Entorhinal cortex (EC)	5776 (0.5%)
Hippocampus (HIP)	5264 (0.5%)
Middle temporal gyrus (MTG)	3379 (0.5%)
Posterior cingulate cortex (PCC)	6536 (0.4%)

Number of common genes across	320
all four DE probe sets	

**Table 2:** Number of genes common to the set of DE genes of the regions being compared and the number of genes with low topological overlap.

Comparison	Number of common	Number of low TO genes
	DE genes	(*P* < 1 × 10*e* ^−135^)
EC-HIP	2041	204
EC-MTG	1398	140
EC-PCC	2424	242
HIP-MTG	1248	125
HIP-PCC	3118	312
PCC-MTG	1582	158

**Table 3:** Significant biological processes of the low TO genes that have a larger number of links in the coexpression network of a particular region.

Number of low TO genes	Number of genes with high connectivity in region	Significant biological processes/pathways
204 EC-HIP	115 genes in EC	Regulation of response to stress
		Cell adhesion-ephrin signaling
		Apoptosis and survival-NO synthesis and signaling
		Cell cycle-chromosome condensation in prometaphase
	95 genes in HIP	Mitochondrial translational termination
		Regulation of protein exit from endoplasmic reticulum
		DNA damage-ATM/ATR regulation of G1/S checkpoint
		Immune response

140 EC-MTG	84 genes in EC	Cell adhesion
		Signal transduction cAMP signaling
		Positive regulation of transcription elongation from RNA polII promoter
		Regulation of transcription involved in G1 phase (mitotic cell cycle)
		Regulation of G2/M transition of mitotic cell cycle
		Apoptosis and survival
	56 genes in MTG	Negative regulation of stress activated MAPK cascade
		Negative regulation of signal transduction
		Activation of astroglial cells proliferation by ACM3
		Notch signaling pathway
		Regulation of lipid metabolism

242 EC-PCC	130 genes in EC	Generation of signal involved in cell-cell signaling
		Positive regulation of mitotic cell cycle
	118 genes in PCC	Proteolysis role of parkin
		Immune response IL6 signaling pathway
		p53 signaling pathway
		Activation of ESR1/SP pathway

125 HIP-MTG	59 genes in HIP	Oxidative phosphorylation
		Neuroligin clustering
		Gephyrin clustering
		Postsynaptic membrane assembly
		Cholesterol and sphingolipids transport
	66 genes in MTG	Transcription
		GTP-XTP metabolism
		ATP/ITP metabolism
		Osmosensory signaling pathway
		Ribonucleotide metabolic process

312 HIP-PCC	145 genes in PCC	Cell adhesion-ephrin signaling
		Immune response
		Negative adaptation of signaling pathway
	174 genes in HIP	Memory and learning
		Regulation of dopamine metabolic process
		Cognition
		Regulation of calcium ion transport
		NMDA-dependent postsynaptic long-term potentiation
		DNA damage, apoptosis, and survival

158 PCC-MTG	83 genes in PCC	Positive regulation of protein tyrosine kinase activity
		Neural plate elongation
		Ubiquinone metabolism
		PEDF signaling
		Cytoskeleton remodeling-RalB regulation pathway
		Antiapoptotic TNFs/NF-kB/IAP pathway
	77 genes in MTG	Glucocorticoid receptor signaling
		Positive regulation of transport

**Table 4:** Disease associations of sets of low TO genes with (extended set) and without (primary set) their direct neighbors in the PPI networks.

Region PPI network	Disease association of low TO genes and neighbors in PPI net	Disease association of low TO genes only
EC-HIP	*Cluster 1 enrichment score = 0.96 *	*Cluster 1 *
	Hypertension	Cardiovascular
	Arterial disease	
	Atherosclerosis	
	Kidney disease	
	Vascular dementia	
	Diabetic nephropathy	

EC-PCC	* Cluster 1 enrichment score = 0.93 *	* Cluster 1 *
	Hyperlipidemia	Ischemic stroke, renal, atherosclerosis
	Coronary artery disease risk	
	Cholesterol	
	* Cluster 2 enrichment score = 0.91 *	* Cluster 2 *
	Heart related conditions	Cancer
	Alzheimer's disease	Macular degeneration

EC-MTG	*Cluster 1 enrichment score = 0.89 *	
	Insulin, cholesterol	
	Type 2 diabetes, hypertension	
	Polycystic ovary syndrome	

HIP-PCC	* Cluster 1 enrichment score = 0.82 *	* Cluster 1 *
	Colon cancer, rectal cancer	Crohn's disease, atherosclerosis, ischemic stroke, renal

PCC-MTG	*Cluster 1 enrichment score = 0.99 *	
	Type 2 diabetes	
	Hypertension	
	Cholesterol	
	Polycystic ovary syndrome	

HIP-MTG	*Cluster 1 enrichment score = 1.23 *	
	Colon cancer, rectal cancer	
	Male infertility	

The low TO genes are proteins in the region PPI network. Column 1 shows the diseases associated with the low TO proteins and their direct neighbors in the regional PPI network while column 2 shows the diseases associated with only the low TO genes (without their neighbors). Disease databases used: OMIM disease, genetic association disease database. Similarity threshold for clustering = 0.80. Results reported only for the clusters with enrichment score 0.8 or higher. In this analysis, the low TO genes were not divided into 2 groups based on which regional coexpression network they had a higher connectivity in.

**Table 5:** Differential connectivity-disease association of the low TO gene modules (low TO proteins and their direct neighbors in the PPI network).

Number of overlapping low TO genes (overlap between coexpression and PPI network nodes)	Number of low TO genes with higher connectivity in region (number of low TO proteins + direct neighbors in module)	Diseases clusters
113 low TO genes in EC-HIP	63 + 5 low TO genes in EC Coexp. net (354 proteins)	Cluster 1 enrichment score = 1.31
		Arterial disease
		Atherosclerosis, generalized blood pressure
		Arterial cardiovascular disease
		Esophageal varices
		Cerebral white matter lesions
		Peritoneal transport
	45 + 5 low TO genes in HIP Coexp. net (304 proteins)	Cluster 1 enrichment score = 0.8
		Stroke, lacunar
		Coronary atherosclerosis
		Thromboembolism, venous
		Stroke, ischemic

94 low TO genes in EC-MTG	39 + 1 low TO genes in MTG Coexp. net (428 proteins)	None
	54 + 1 low TO genes in EC Coexp. net (325 proteins)	Cluster 1 enrichment score = 1.15
		Nephropathy
		Stroke
		Restenosis
		Cluster 2 enrichment score = 0.98
		Panencephalitis, subacute sclerosing
		Sarcoidosis; tuberculosis
		Tuberculosis
		Hepatitis B

126 low TO genes in EC-PCC	68 + 3 low TO genes in EC Coexp. net (405 proteins)	Cluster 1 enrichment score = 0.88
		Ischemia
		Hyperlipidemia
		Lipids
		Hypercholesterolemia
	55 + 3 low TO genes in PCC Coexp. net (468 proteins)	None

76 low TO genes in HIP-MTG	36 + 0 low TO genes in HIP Coexp. net (213 proteins)	None
	40 + 0 low TO genes in MTG Coexp. net (288 proteins )	None

171 low TO genes in HIP-PCC	94 + 3 low TO genes in HIP Coexp. net (552 proteins)	Cluster 1 enrichment score = 1.31
		Colon cancer; rectal cancer
		Androgen levels
		Hypospadias
	74 + 3 low TO genes in PCC Coexp. net (413 proteins)	Cluster 1 enrichment score = 1.31
		Coronary atherosclerosis
		Glomerulonephritis
		Cerebrovascular disease

87 low TO genes in PCC-MTG	44 + 1 low TO genes in MTG Coexp. net (644 proteins)	None
	42 + 1 low TO genes in PCC Coexp. net (357 proteins)	Cluster 1 enrichment score = 0.82
		Mood disorder
		Smoking behavior
		Alcoholism
		Suicide

This table provides the diseases linked with differentially connected low TO genes and direct neighbors in the PPI net. Each comparison has the following format: number of low TO genes with higher connectivity in regional Coexp. net = number of low TO genes with higher connectivity in one brain region + number of low TO genes with equal connectivity in both brain regions. For example, in the 113 EC-HIP comparison there are 113 low TO genes of which 63 genes have a higher connectivity in the EC compared to HIP coexpression network, and in addition 5 of them have equal number of connections in both coexpression networks, whereas 45 genes have a higher connectivity in the HIP with 5 extra genes having equal connectivity in both regions. The number of proteins and direct neighbors in the PPI net, that is, the module, is given in brackets. In EC HIP, 204 low TO genes ⋂‍ respective pruned PPI net = 113 proteins. In EC MTG, 140 low TO genes ⋂‍ respective pruned PPI net = 94 proteins. In EC PCC, 242 low TO genes ⋂‍ respective pruned PPI net = 126 proteins. In HIP MTG, 125 low TO genes ⋂‍ respective pruned PPI net = 76 proteins. In HIP PCC, 312 low TO genes ⋂‍ respective pruned PPI net = 171 proteins. In PCC MTG, 158 low TO genes ⋂‍ respective pruned PPI net = 87 proteins. This table reveals that MTG is not cardiovascular disease (CVD) associated. When comparing HIP and PCC, PCC is highly associated with CVD, while HIP is not. When comparing EC and PCC, EC is highly associated with CVD, while PCC is not. When comparing EC and HIP, both regions are associated with CVD. When comparing EC and MTG, EC is associated with CVD, while MTG is not. Overall, EC is the brain region mostly associated with CVD. HIP and PCC follow, and PCC might be more active in CVD than HIP. MTG is least associated with CVD of all four brain regions.

**Table 6:** Disease association of the pairs of low TO gene modules in Soler-López et al.'s AD PPI network.

Number of overlapping low TO genes	Number of low TO genes with high connectivity in region (number of proteins in low TO modules)	Enriched disease clusters
15 EC-HIP	9 + 1 low TO genes in EC (67 proteins)	Cluster 1 enrichment score = 1.88
		Arterial disease
		Atherosclerosis, generalized blood pressure
		Arterial cardiovascular disease
		Cerebral white matter lesions
		Esophageal varices
		Peritoneal transport
	5 + 1 low TO genes in HIP (35 proteins)	None

21 EC-MTG	12 + 0 low TO genes in EC (60 proteins)	None
	9 + 0 low TO genes in MTG (125 proteins)	None

23 EC-PCC	12 + 0 low TO genes in EC (66 proteins)	None
	11 + 0 low TO genes in PCC (88 proteins)	Cluster 1 enrichment score = 0.98
		Coronary atherosclerosis
		Lipoprotein
		Cardiovascular disease
		Myocardial infarction
		Cluster 2 enrichment score = 0.88
		Lipoprotein
		Myocardial infarction
		Coronary artery disease
		Atherosclerosis, coronary

12 HIP-MTG	5 + 0 low TO genes in HIP (18 proteins)	Cluster 1 enrichment score = 0.9
		Prostate cancer
		Breast cancer
		Pharmacogenomic
		Cancer
	7 + 0 low TO genes in MTG (37 proteins)	None

27 HIP-PCC	12 + 1 low TO genes in PCC (58 proteins)	Cluster 1 enrichment score = 1.58
		Coronary artery disease
		Stroke
		Crohn's disease ulcerative colitis
		Restenosis
		Cluster 2 enrichment score = 0.83
		Melanoma
		Stomach cancer
		Asthma
	14 + 1 low TO genes in HIP (95 proteins)	None

16 PCC-MTG	6 + 1 low TO genes in PCC (47 proteins)	None
	9 + 1 low TO genes in MTG (112 proteins)	None

Number of low TO genes with high connectivity in regional coexpression network = number of low TO genes with higher connectivity in one brain region + number of low TO genes with equal connectivity in both brain regions. There are 1704 proteins in ADnet. In EC HIP, 204 low TO genes ⋂‍ ADnet = 15 proteins. In EC MTG, 140 low TO genes ⋂‍ ADnet = 21 proteins. In EC PCC, 242 low TO genes ⋂‍ ADnet = 23 proteins. In HIP MTG, 125 low TO genes ⋂‍ ADnet = 12 proteins. In HIP PCC, 312 low TO genes ⋂‍ ADnet = 27 proteins. In PCC MTG, 158 low TO genes ⋂‍ ADnet = 16 proteins. Disease annotation cluster analysis was conducted on pairs of exclusive low TO modules (pairs of low TO modules excluding common proteins between module pairs). MTG seems to be least associated with cardiovascular diseases (CVD). When comparing EC and HIP, EC is highly associated with CVD. When comparing EC and PCC, PCC is highly associated with CVD. When comparing HIP and PCC, PCC is highly associated with CVD.

**Table 7:** Comparison of mean eigenvector centrality scores (*A* *v* *EgC*) of low TO genes in control and affected coexpression networks per region.

Region	EC	PCC	HIP	MTG
*Av* *Eg* *C*	Affected < control	Affected < control	Affected < control	Affected > control

*P* value	1.18*e* − 3	7.10*e* − 9	7.77*e* − 9	3.05*e* − 1

Low TO genes were identified between the control and affected networks in each of the 4 brain regions. The eigenvector centrality scores of these low TO genes in the control and affected networks were calculated for each region and their means (*A* *v* *EgC*) were compared. Statistical significance is reached if *P* value is <0.05.

**Table 8:** Comparison of mean eigenvector centrality scores (*A* *v* *EgC*) of DE genes in control and affected coexpression networks per region.

Region	EC	PCC	HIP	MTG
*Av* *Eg* *C*	Affected < control	Affected < control	Affected < control	Affected < control

*P* value	6.39*e* − 1	2.60*e* − 1	2.57*e* − 6	1.80*e* − 12

Comparison of the average eigenvector centrality scores (*A* *v* *EgC*) of the differentially expressed genes within each region. Statistical significance is reached if *P* value is <0.05. In all cases, the mean eigenvector score of the affected networks was smaller than that of the control networks, although it was only statistically significant in the hippocampus and middle temporal gyrus.

**Table 9:** Comparison of mean eigenvector centrality scores (*EgC*_*e*_) of low TO genes between brain regions.

*Eg* *C*_*e*_	EC < PCC	EC < HIP	EC < MTG	PCC < HIP	PCC < MTG	HIP < MTG
*P* value	9.50*e* − 6	1.22*e* − 7	2.20*e* − 16	1.42*e* − 1	2.15*e* − 12	6.331*e* − 9

Comparison of the average eigenvector centrality scores of the low TO genes between regions. Statistical significance is reached if *P* value is <0.05.
